# Amiloidosis Cardíaca: Experiencia en un Instituto Cardiovascular de Referencia Nacional

**DOI:** 10.47487/apcyccv.v1i2.40

**Published:** 2020-06-29

**Authors:** Juan Muñoz-Moreno, José Añorga-Ocmin, Sandra Espinola-García, Cristian Aguilar-Carranza, Walter Alarco-León

**Affiliations:** 1 Médico residente de Cardiología. Instituto Nacional Cardiovascular - INCOR EsSalud. Lima, Perú. Instituto Nacional Cardiovascular - INCOR EsSalud Lima Perú; 2 Médico asistente del Laboratorio de Patología. Instituto Nacional Cardiovascular INCOR EsSalud. Lima, Perú. Instituto Nacional Cardiovascular INCOR EsSalud Lima Perú; 3 Unidad de Insuficiencia Cardíaca, Trasplante Cardíaco e Hipertensión Pulmonar. Lima, Perú. Unidad de Insuficiencia Cardíaca, Trasplante Cardíaco e Hipertensión Pulmonar Lima Perú

**Keywords:** amiloidosis, insuficiencia cardíaca, Perú, amyloidosis, heart failure, Peru

## Abstract

**Objetivos:**

Determinar las características clínicas, de imágenes y laboratoriales así como la sobrevida al año del diagnóstico de pacientes con amiloidosis cardíaca en un hospital de referencia nacional.

**Materiales y métodos:**

Estudio de serie de casos. Evaluamos las características clínicas, exámenes complementarios y supervivencia de pacientes con amiloidosis cardíaca diagnosticados, tratados y seguidos en el servicio de Cardiología Clínica del Instituto Nacional Cardiovascular - INCOR EsSalud.

**Resultados:**

Se encontraron 8 pacientes con diagnóstico de amiloidosis cardíaca. La mediana de la edad fue 64.5 años y el 75% de sexo masculino. La etiología fue amiloidosis cardíaca no especificada (25%), amiloidosis cardíaca transtiretina (37.5%) y amiloidosis cardíaca de cadenas ligeras (37.5%). La insuficiencia cardíaca sintomática (NYHA II-III) fue la presentación inicial más común (87.5%). Las manifestaciones extracardíacas más frecuentes fueron: neuropatía sensitivo-motora (62.5%), musculoesqueléticas (37.5%), nefropatía (25%), síndrome de túnel carpiano bilateral (25%), gammapatías monoclonales (25%) y efusión pleural refractaria (25%). La sobrevida al año fue del 75% y la causa de muerte en los 2 fallecidos fue muerte súbita.

**Conclusiones:**

En este estudio sobre amiloidosis cardíaca en un centro especializado las manifestaciones clínicas más frecuentes fueron la insuficiencia cardíaca y la neuropatía sensitivo-motora. La mortalidad fue del 25% al año, y en todos los casos como muerte súbita.

## Abstract (en)

**Objectives:**

To determine the clinical, imaging and laboratory characteristics and one year after diagnosis survival of patients with cardiac amyloidosis in a national reference hospital.

**Materials and methods:**

Case series study. We evaluated the clinical characteristics, complementary examinations and survival of patients with cardiac amyloidosis diagnosed, treated and followed up in the Clinical Cardiology service of the National Cardiovascular Institute - INCOR EsSalud in Lima, Peru.

**Results:**

We found eight patients with diagnosis of cardiac amyloidosis. The median age was 64.5 years and 75% were male. The etiology of cases was unspecified cardiac amyloidosis (25%), transthyretin cardiac amyloidosis (37.5%), and light chain cardiac amyloidosis (37.5%). Symptomatic heart failure (NYHA II-III) was the most common initial presentation symptom (87.5%). The most frequent extracardiac manifestations were: sensory-motor neuropathy (62.5%), musculoskeletal (37.5%), nephropathy (25%), bilateral carpal tunnel syndrome (25%), monoclonal gammopathies (25%) and refractory pleural effusion (25 %). Survival at one year was 75% and the cause of the 2 deaths was sudden death.

**Conclusions:**

In this study of cardiac amyloidosis at a specialized center, the most frequent clinical manifestations were heart failure and sensory-motor neuropathy. Mortality was 25% per year, and in all cases as sudden death.

## Introducción

La amiloidosis es una condición clínica, caracterizada por el depósito extracelular de fibrillas de amiloide, las cuales son proteínas con una estructura terciaria inestable. ^(^^1^^,^^2^^)^ Cuando el depósito primario ocurre en el espacio intersticial del corazón y no está asociado con patología de los cardiomiocitos, se le denomina amiloidosis cardíaca. ^(^^1^^)^ La clasificación de la amiloidosis se basa en la proteína precursora. Por ejemplo: la producción clonal de cadenas ligeras de inmunoglobulinas mal plegadas resulta en la amiloidosis de cadenas ligeras (AL), mientras que el depósito anormal de transtiretina causa la amiloidosis transtiretina (ATTR) y juntas representan aproximadamente el 95% de los casos de amiloidosis cardíaca. ^(^^2^^,^^3^

En amiloidosis AL, el riñón es el órgano más afectado, seguido por el corazón, y es la extensión de este último lo que determina el pronóstico. ^(^^1^^,^^4^^,^^5^^)^ Por otro lado, la amiloidosis ATTR puede manifestarse como un trastorno focalizado (cardíaco) o sistémico. Existen 2 subtipos de amiloidosis ATTR, la amiloidosis hereditaria o mutante (ATTRm) secundaria a mutaciones del gen trantiretina (TTR) y la de forma natural o salvaje (ATTRwt) originada por cambios en la estabilidad de TTR relacionados con la edad. ^(^^6^^)^ Se estima una prevalencia de 5.5% a 16% en mayores de 80 años para la ATTRwt, en comparación con la ATTRm que es menos frecuente. ^(^^2^^)^

No tenemos reportes de casos de amiloidosis cardíaca en Perú, por lo que nuestro objetivo es determinar las características clínicas, de imágenes, y laboratoriales así como la sobrevida al año del diagnóstico, de los pacientes con amiloidosis cardíaca en un hospital de referencia nacional.

## Material y Método

Se realizó un estudio de serie de casos de pacientes con diagnóstico de amiloidosis cardíaca en el servicio de Cardiología Clínica del Instituto Nacional Cardiovascular - INCOR EsSalud desde el año 2014 hasta el año 2020, obteniendo los registros de las historias clínicas donde se evaluaron características clínicas, de imágenes, laboratoriales, tratamiento y sobrevida al año.

Las variables cualitativas se expresaron en frecuencias y porcentajes, mientras que las cuantitativas en mediana y rango intercuartil (RIQ) al ser variables que no siguen una distribución normal. El análisis de sobrevida al año, se hizo evaluando la relación porcentual entre el número de casos vivos y el total de casos. Para procesar la información se utilizó el programa estadístico Microsoft Excel versión 16.33 - 2019.

## Resultados

Se encontraron 8 pacientes con diagnóstico de amiloidosis cardíaca. El 75% de los pacientes fue de sexo masculino, la mediana de la edad fue 64.5 años (RIQ: 46.5 - 82.5). La etiología de los casos fue: amiloidosis cardíaca no especificada (25%), del tipo transtiretina (37.5%) y de cadenas ligeras (37.5%). Los casos donde no se especificó el subtipo de amiloide, se debió a la falta de disponibilidad de gammagrafía e inmunohistoquímica al momento del diagnóstico. (Tabla 1)

**Tabla 1:** Características de los pacientes

Número de Caso	Edad	Sexo	Fecha de Diagnóstico (mes/año)	Examen diagnóstico		Diagnóstico
				Inicial	Definitivo	
1	56	M	11/2014	ETT	BEM*, Estudio Genético*	ATTRm Val30Met
2	60	F	02/2015	ETT	BEM	AC no especificada
3	59	F	06/2016	ETT	BEM	AC no especificada
4	69	M	05/2019	ETT, RMC*, Dosaje CLLs, EFIs*, EFIu*	Gammagrafía	ATTR
5	56	M	05/2019	ETT, RMC*, Dosaje CLLs, EFIs*, EFIu*, Gammagrafía	BEM, IHQ	AL (cadenas ligeras kappa)
6	75	M	08/2019	ETT, RMC*, Dosaje CLLs	BEM, IHQ	AL (cadenas ligeras kappa)
7	78	M	11/2019	ETT, Dosaje CLLs*, EFIs*, EFIu*	BP*, IHQ*	AL (cadenas ligeras kappa)
8	76	M	12/2019	ETT, RMC*, Dosaje CLLs, EFIs*, EFIu*	Gammagrafía	ATTR

M: masculino; F: femenino; AC: amiloidosis cardíaca; AL: amiloidosis de cadenas ligeras; ATTR: amiloidosis transtiretina; ATTRm: amiloidosis transtiretina por mutación hereditaria; BEM: biopsia endomiocárdica; IHQ: inmunohistoquímica; BP: biopsia pleural; ETT: ecocardiograma transtorácico; RMC: resonancia magnética cardíaca; CLLs: cadenas ligeras libres séricas; EFIs: electroforesis e inmunofijación sérica; EFIu: electroforesis e inmunofijación en orina.

* Examen realizado en otra institución.

### Manifestaciones clínicas

**Figura 1:**
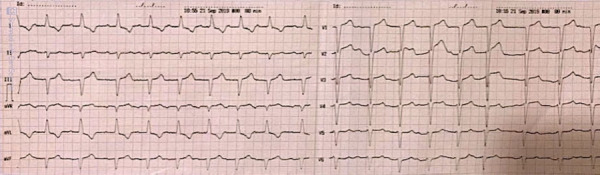
Electrocardiograma de 12 derivadas muestra trastorno de conducción (bloqueo auriculoventricular de primer grado), patrón de pseudoinfarto con ondas Q en derivadas III, aVF, V1-V2, y ausencia de progresión de ondas R en precordiales (caso 4).

La insuficiencia cardíaca sintomática (New York Heart Association (NYHA) II-III) fue la presentación inicial más común (87.5%), y en el seguimiento se manifestó en el 100% de los pacientes. Además, el 37.5% presentó angina y el 25% síncope. Las manifestaciones extracardíacas más frecuentes fueron: neuropatía sensitivo-motora (62.5%), musculoesqueléticas (37.5%), nefropatía (25%), síndrome de túnel carpiano bilateral (25%), gammapatías monoclonales (25%) y efusión pleural refractaria (25%). Los hallazgos electrocardiográficos más frecuentes fueron el patrón de pseudoinfarto (37.5%), ausencia de progresión de R en precordiales (25%) y bloqueo auriculoventricular de primer grado (25%). (Figura 1)

### Hallazgos laboratoriales

Los marcadores de mal pronóstico estudiados fueron: propéptido natriurético cerebral (ProBNP), que siempre se encontró elevado (promedio 11 200 pg/ml); troponina T, que en el 71.4% de pacientes a los que se les realizó el examen tu-vo un valor ≥ 0.05ng/ml; y tasa de filtración glomerular, que en el 62.5% de los casos fue menor a 45 ml/min/1.73m^2^. (Tabla 2)

**Tabla 2:** Características clínicas y de laboratorio, tratamiento y supervivencia

	Número de Caso							
	1	2	3	4	5	6	7	8
Características Clínicas								
Clínica de presentación	IC - Disnea CF II	TPSV, STC	IC - Disnea CF III	IC - Disnea CF II	IC - Disnea CF III	IC - Disnea CF III	IC - Disnea CF III	IC - Disnea CF II
Insuficiencia cardíaca (IC)	Sí	Sí	Sí	Sí	Sí	Sí	Sí	Sí
Angina	Sí	No	No	No	No	Sí	Sí	No
Síncope	Sí	No	No	Si	No	No	No	No
Estenosis aórtica	No	No	No	No	No	No	No	No
Manifestaciones extracardíacas	Disautonomía,^a^ nefropatía, neuropatía sensitivo-motora^b^	STC bilateral, neuropatía sensitivo-motora,^b^ músculo esqueléticas^c^	Afección ocular,^d^ nefropatía, músculo esqueléticas^c^	STC bilateral, estenosis lumbar, nefropatía	MM, EP refractaria,^e^ nefropatía, neuropatía sensitivo-motora^b^	Músculo esqueléticas,^c^ nefropatía, neuropatía sensitivo-motora^b^	MW, EP refractaria,^e^ nefropatía, neuropatía sensitivo-motora,^b^ AIT	No
Comorbilidades	PO CMHO (2014), Portador de MCP bicameral (2018)	HTA, PO STC derecho (2014)	Ninguno	HTA	DM, HTA	Ninguno	DM, hipotiroidismo	HTA, PO CVAm (2008)
Hallazgos electrocardiográficos	FA	TRNAV	Patrón de pseudoinfarto	Patrón de pseudoinfarto, ausencia de progresión de R en precordiales, BAV I grado	Complejos de bajo voltaje, patrón de pseudoinfarto, ausencia de progresión de R en precordiales	BAV I grado	FA	Normal
Laboratorio								
ProBNP (pg/ml)	4855	(-)	(-)	26 381	15 242	2338	7187	(-)
Troponina T (ng/ml)	0.18	0.01	0.121	0.83	0.082	0.045	0.177	(-)
TFG (ml/min/1.73 m2)	37.13	78.7	41.27	33.95	43.48	49.87	40.23	61.97
Tratamiento								
Inicial	Tafamidis, doxiciclina, diuréticos, NACO	BB, ARA II, diuréticos, gabapentina	Diuréticos	Diuréticos	Diuréticos, IECA, BB, bortezomib, talidomida, pamidronato, dexametasona	BB, diuréticos	Diuréticos, rituximab, bortezomib, ciclofosfamida, dexametasona	BB, ARA II, diuréticos, ACO
Actual	TH, prednisona, tacrolimus, TMP/SMX, gabapentina	NA	NA	Diuréticos, doxicilina y ACUD	NA	BB, diuréticos	Diuréticos, gabapentina, NACO	BB, ARA II, diuréticos, ACO
Supervivencia								
Sobrevida desde el diagnóstico	5 años 6 meses	1 año 1 mes	3 meses	1 año	8 meses	9 meses	6 meses	5 meses
Estado vital actual	Vivo	Fallecido	Fallecido	Vivo	Fallecido	Vivo	Vivo	Vivo
Causa de muerte	NA	Muerte súbita	Muerte súbita	NA	Muerte súbita	NA	NA	NA

TPSV: taquicardia paroxística supraventricular; STC: síndrome del túnel carpiano; EP: efusión pleural; MM: mieloma múltiple; MW: macroglobulinemia de Waldenström; AIT: ataque isquémico transitorio; CMHO: cardiomiopatía hipertrófica obstructiva; MCP: marcapaso; HTA: hipertensión arterial; PO: postoperado; DM: diabetes mellitus; CVAm: cambio de válvula aórtica mecánica; FA: fibrilación auricular; TRNAV: taquicardia por reentrada del nodo auriculoventricular; BAV: bloqueo auriculoventricular; FA: fibrilación auricular; TFG: tasa de filtración glomerular; (-): examen no solicitado; NACO: nuevo anticoagulante oral; BB: beta bloqueador; ARA II: antagonista del receptor de angiotensina II; IECA: inhibidor de enzima convertidora de angiotensina; ACO: anticoagulación oral; TH: trasplante hepático; TMP/SMX: trimetoprim/sulfametoxazol; NA: no aplica; ACUD: ácido ursodesoxicólico.

a) Disautonomía: hipotensión ortostática, gastroparesia, disfunción eréctil, vejiga neurogénica. b) Neuropatía sensitivo-motora: parestesias, paraparesia de miembros inferiores. c) Músculo esqueléticas: pérdida de peso, lumbalgia, fatiga, debilidad muscular. d) Afectación ocular: depósitos intravítreos, visión borrosa. e) EP refractaria: efusión pleural del lado derecho, refractaria a toracocentesis.

### Hallazgos en las imágenes

La hipertrofia del ventrículo izquierdo (VI), el engrosamiento del septum interventricular y pared posterior del VI, (Figura 1A) disfunción diástolica grado III y algún grado de insuficiencia mitral fueron encontrados en el 100% de casos. La fracción de eyección del VI (FEVI) fue preservada, intermedia y reducida en el 37.5%, 50% y 12.5% respectivamente; con una mediana de 48.5% (RIQ: 40.5 - 56.5). (Tabla 3) Otros hallazgos ecográficos se muestran en la Figura 1B. Además, se realizó strain longitudinal al 75% de los pacientes, encontrándose en todos el patrón típico de “ojo de buey”. (Figura 1C y 1D) (Video 1)

**Tabla 3:** Ecocardiograma transtorácico

	N = 8	
Fracción de eyección de VI	48	(40.5 -56.5)
Fracción de eyección de VI preservada	3	(37.5)
Fracción de eyección de VI intermedia	4	(50.0)
Fracción de eyección de VI reducida	1	(12.5)
Cambio de área fraccional de VD	35	(27.5 - 45.5)
Hipertrofia de VI	8	(100.0)
Hipertrofia de VD	3	(37.5)
Engrosamiento del SIV	8	(100.0)
Engrosamiento de PP del VI	8	(100.0)
Engrosamiento del septum interatrial	3	(37.5)
Dilatación biatrial	4	(50.0)
Disfunción diastólica grado III	8	(100.0)
Insuficiencia mitral	8	(100.0)
Leve	1	(12.5)
Moderada	4	(50.0)
Severa	3	(37.5)
Insuficiencia tricuspídea	7	(87.5)
Leve	3	(37.5)
Moderada	2	(25.0)
Severa	2	(25.0)
Baja probabilidad de HTP	5	(62.5)
Alta probabilidad de HTP	3	(37.5)
Derrame pericárdico leve	5	(8.7)

Se reporta mediana (rango intercuartil) y frecuencias (porcentaje) para variables cuantitativas y categóricas, respectivamente.

VI: ventrículo izquierdo; VD: ventrículo derecho; SIV: septum interventricular; PP: pared posterior; HTP: hipertensión pulmonar.

Los patrones característicos de amiloidosis cardíaca en la resonancia magnética cardíaca pueden ser patrones subendocárdicos o transmurales. (Video 2) Estos y los hallazgos confirmatorios realizados en el estudio anatomo-pa-tológico se pueden observar en las Figuras 3 y 4 respectivamente. En la gammagrafía con tecnesio 99m pirofosfato, mostramos la diferencia de captación del miocardio entre un paciente con ATTR (grado 3) y AL (grado 1). (Figura 5)

**Figura 2:**
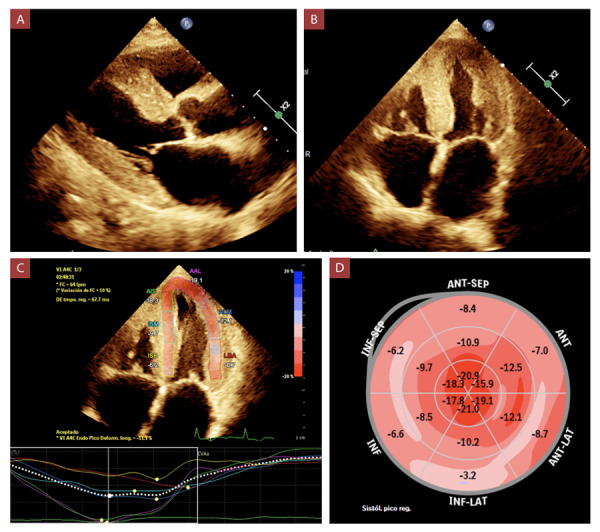
Ecocardiograma transtorácico correspondiente al caso 6. **A)** Vista paraesternal eje largo que muestra engrosamiento del septum interventricular y pared posterior del ventrículo izquierdo. **B)** Vista apical 4-cámaras que muestra engrosamiento de las paredes de ambos ventrículos, del septum interventricular e interatrial, dilatación biatrial, y engrosamiento de los velos de las válvulas mitral y tricúspide. **C)** Strain longitudinal global reducido (-11), con valores conservados en ápex y reducción a nivel basal y medio. **D)** Mapa polar que muestra todos los segmentos del ventrículo izquierdo con patrón característico en “ojo de buey”.

**Figura 3:**
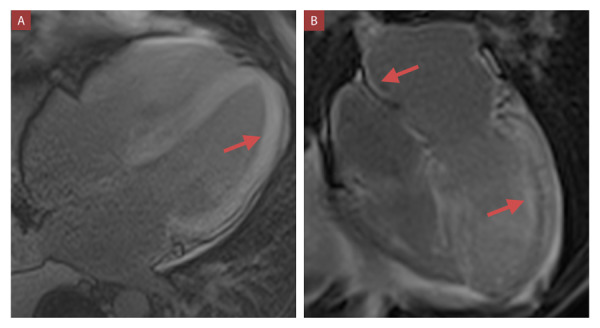
Resonancia magnética cardíaca en vista apical 4 cámaras que muestra captación con realce tardío de gadolinio (flecha roja): **A)** Patrón transmural ventricular. (Caso 5) **B)** Patrón subendocárdico difuso biventricular, del septum interauricular y de las paredes auriculares (flechas rojas). Además se observa la anulación del pool sanguíneo. (Caso 6)

**Figura 4:**
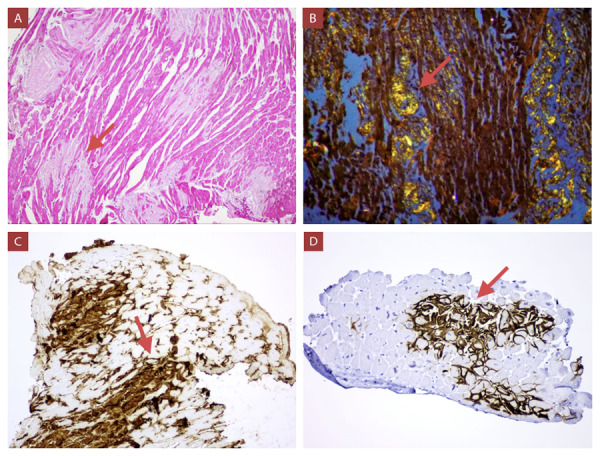
Biopsias endomiocárdicas. **A)** Depósito difuso de material amorfo, eosinofílico, de patrón nodular (flecha roja; HE, 10x, Caso 6). **B)** Birrefringencia “verde manzana” (flecha roja) a la luz polarizada (Rojo Congo, 10X, Caso 3). **C)** Depósito intersticial de tendencia nodular positivo para cadena ligera Kappa (flecha roja; IQH, 20X, Caso 6). **D)** Depósito intersticial positivo para transtiretina (flecha roja; IQH, 20X, Control).

**Figura 5:**
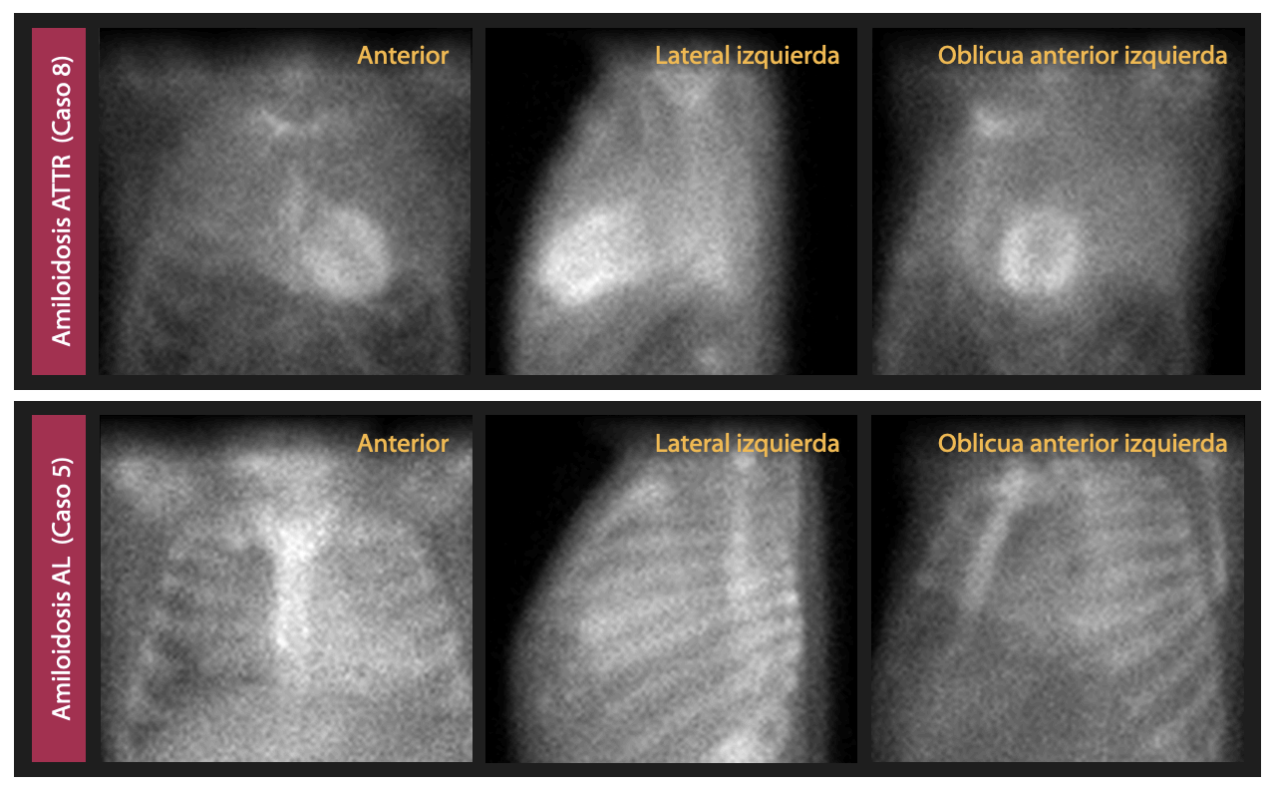
Gammagrafías con tecnesio 99m pirofosfato de 2 pacientes: La primera corresponde a amiloidosis cardíaca transtiretina (ATTR), (caso 8) y permite observar la captación biventricular y superior al hueso, correspondiente con grado 3 (grave) de Perugini. La segunda es compatible con amiloidosis de cadenas ligeras (AL), (caso 5) y muestra captación menor al hueso, es decir, grado 1 (leve).

### Tratamiento y sobrevida

2 casos de amiloidosis AL recibieron quimioterapia: El caso 7 presentó macroglobulinemia de Waldenström, por lo cual recibió quimioterapia y rituximab, pero un mes después se suspendió por descompensación de la insuficiencia cardíaca. El caso 5 presentó mieloma múltiple, por lo que también recibió quimioterapia. (Tabla 2)

El caso 1 con amiloidosis ATTRm Val30Met, recibió tafamidis y doxiciclina; además se le implantó un marcapaso bicameral, por la presencia de fibrilación auricular con frecuencia lenta, que incrementaba la frecuencia de cuadros sincopales debido a disautonomía. Se le realizó trasplante de hígado y es el paciente con la mayor supervivencia en nuestro estudio (5 años y 6 meses).

La menor sobrevida desde el diagnóstico fue de 3 meses en un caso de amiloidosis cardíaca no especificada y de 8 meses en AL. La sobrevida al año fue del 75%. La causa de muerte en el 100% de los casos fallecidos fue muerte súbita. (Tabla 2)

## Discusión

Los trabajos nacionales publicados hasta la fecha han sido reportes de casos aislados. Esta es la primera serie de casos de amiloidosis cardíaca en nuestro país, que además cuenta con resultados de resonancia magnética cardíaca, biopsia, gammagrafía cardíaca, entre otros estudios, para alcanzar el diagnóstico preciso. 

La mayoría de nuestros pacientes tenía una edad mayor a 60 años al momento del diagnóstico, tal como lo reportan Fine *et al.* (2020), con preponderancia en el sexo masculino. ^(^^7^

Povar *et al.* (2020) realizaron un estudio en 30 pacientes con amiloidosis cardíaca, y encontraron que la insuficiencia cardíaca estuvo presente en el 93.3%.^8^^)^ En nuestro estudio, la presentación inicial más frecuente fue insuficiencia cardíaca, lo que destacamos, ya que en este contexto la supervivencia sin tratamiento se reduce a menos de 6 meses en amiloidosis cardíaca tipo AL, que es más agresiva y frecuente que la amiloidosis ATTR. ^(^^9^^,^^10^^)^ El diagnóstico tardío, reflejado en la presencia de insuficiencia cardíaca, podría deberse a la variedad en las manifestaciones clínicas y que la amiloidosis es una entidad de baja sospecha, por la escasa experiencia en la integración de la clínica con los exámenes complementa-rios, considerando que son pocos los casos en la práctica diaria.

Entre las manifestaciones clínicas que sugieren amiloidosis AL, Isabel *et al.* (2013) reportaron la presencia de angina en 8.3%, mientras que en nuestro estudio estuvo presente en el 66.7% de los casos. ^(^^11^^)^ La angina se produce por infiltración de amiloide alrededor de los vasos intramiocárdicos sin mayor compromiso de las arterias epicárdicas. ^(^^12^^)^ Un examen neurológico completo es importante, dado que la neuropatía sensitivo-motora se reporta en la literatura entre 25 y 33%, y en nuestro estudio estuvo presente en el 100% de los casos. ^(^^11^^,^^13^^)^ Uno de los pacientes con amiloidosis AL presentó mieloma múltiple y otro desarrolló macroglobulinemia de Waldenström, ambos casos acompañados de efusión pleural derecha refractaria a toracocentesis; esto se produce porque la pleura parietal está infiltrada de amiloide que inhibe la absorción de líquido pleural, tal como lo reporta Berk *et al.* (2003) en una serie de pacientes con amiloidosis AL y efusión pleural refractaria. ^(^^14^^,^^15^^)^ En amiloidosis AL, es común encontrar pacientes con insuficiencia cardíaca con fracción de eyección preservada, gammapatía monoclonal de significado incierto (macro-globulinemia de Waldenström o mieloma múltiple), síndrome nefrótico y neuropatía periférica. ^(^^9^^)^ Todos estos ha-llazgos fueron encontrados en nuestro estudio.

En el caso de amiloidosis ATTRm existen manifestaciones clínicas características, como la hipotensión ortostática, gastroparesia y disfunción eréctil, tal como lo reportan Fine *et al.* (2020), ^(^^7^^)^ y se refleja en nuestro caso 1 que presentó disautonomía. González *et al.* (2017) describen que la estenosis aórtica degenerativa, síndrome de túnel carpiano (33-49%) y estenosis lumbar son características de la amiloidosis ATTRwt. ^(^^16^^)^ De acuerdo a ello, planteamos que los casos 4 y 8 diagnosticados por gammagrafía de amiloidosis ATTR, podrían tratarse del subtipo ATTRwt. 

El hallazgo electrocardiográfico más frecuente fue el patrón de pseudoinfarto, definido por Murtagh *et al.* (2005) como ondas QS en 2 derivaciones consecutivas, y reportado en 47% de sus casos, de igual manera por Gonzales *et al*.(2017) en 63-66%.^16^^,^^17^^)^ La presencia de fibrilación auricular es frecuente por dilatación progresiva de las aurículas y la fisiología restrictiva, y se asocia a una alta incidencia de eventos tromboembólicos, como en el caso 7 que presento un ataque isquémico transitorio. ^(^^12^

Las características de la amiloidosis cardíaca en el ecocardiograma incluyen ventrículos pequeños, aumento en el grosor de la pared de VI (> 12 mm), el cual es simétrico en amiloidosis AL o asimétrico en amiloidosis ATTR. ^(^^18^^)^ Asimismo el patrón restrictivo es típico pero generalmente aparece en estadios avanzados de la enfermedad, lo que implica que nuestros casos tuvieron un diagnóstico tardío ya que el 100% tuvo disfunción diastólica grado III. ^(^^19^^,^^20^^)^ Además, es frecuente que la amiloidosis cardíaca se presente como insuficiencia cardíaca con fracción de eyección del ventrículo izquierdo (FEVI) preservada en estadios tempranos. ^(^^18^^)^ En nuestro estudio los casos de FEVI preservada fueron del 37.5%. 

La evaluación del strain longitudinal mediante Doppler tisular muestra preservación de los segmentos apicales con deterioro severo en los segmentos medio y basal (patrón “ojo de buey”). Este hallazgo tiene una sensibilidad de 93% y especificidad de 82%.^18^^,^^19^^,^^20^^)^ En nuestro estudio al 75% se le realizó strain longitudinal, y se encontró el patrón típico de “ojo de buey” en todos los casos. En la resonancia magnética cardíaca con realce tardío de gadolinio se puede evidenciar patrones típicos subendocárdicos difusos, característico de estadios tempranos, y patrones transmurales típicos de estadios posteriores, que presentan peor prónostico evolutivo. ^(^^18^^,^^21^^)^

La gammagrafía con Tc-99m pirofosfato posee un rol central en el diagnóstico no invasivo de la amiloidosis cardíaca, con una sensibilidad mayor a 99% y especificidad de 86% para amiloidosis ATTR. La cuantificación de la captación miocárdica de Tc-99m pirofosfato positivo para ATTR, se realiza de dos formas: por medio del índice de captación cuantitativa miocardio/pulmón (H/CL) contralateral a la hora de tomado el examen con un valor ≥1.5, o por medio de la comparación visual con la captación de las costillas (semicuantificación) a las 3 horas, con una puntuación ≥ 2 en imágenes planares. ^(^^22^^,^^23^^)^ Para la evaluación visual se emplea la clasificación de Perugini, donde grado 0 significa ninguna captación, grado 1 captación menor que las costillas, grado 2 igual que las costillas y grado 3 mayor que las costillas. ^(^^21^^,^^22^^)^ Es importante tener en cuenta, que cuando se asocian una puntuación semicuantitativa de Grado ≥ 2 con la ausencia de proteínas monoclonales en sangre y orina, se obtiene una especificidad y valor predictivo positivo de 100% para el diagnóstico de amiloidosis ATTR. ^(^^3^^,^^23^^)^ En nuestro estudio los casos 4 y 8 presentaron una captación de grado 2 y 3 respectivamente, y luego de descartar la presencia de proteínas monoclonales, fueron diagnosticados de amiloidosis ATTR. Por el contrario, en el caso 5 al evidenciarse una captación de grado 1, y la presencia de proteínas en suero y orina (cadenas ligeras kappa), se realizó una biopsia endomiocardica con inmunohistoquímica, diagnosticándose amiloidosis AL.

La anatomía patológica, además de los hallazgos en las tinciones hematoxilina/eosina y rojo congo, dispone de inmunohistoquímica y análisis genético. El estudio genético es la única forma de distinguir la amiloidosis ATTRm de la ATTRwt. La constatación de una mutación causal como en el caso 1, permite ofrecer una terapia dirigida y además la monitorización de portadores asintomáticos en familiares. ^(^^12^^,^^24^

Existen marcadores de laboratorio importantes en amiloidosis cardíaca. En amiloidosis AL, el NT-pro-BNP (>1,800pg/mL) y la troponina T (> 0.03ng/mL), son marcadores de mal pronóstico. ^(^^9^^)^ En amiloidosis ATTR cambian los valores de NT-proBNP (> 3.000 pg/ml), troponina T (> 0,05 ng/ml) y Gillmore *et al.*, incorpora la tasa de filtración glomerular < 45 ml/min/1.73m^2^. ^(^^16^^,^^25^^,^^26^^,^^27^^)^ En todos nuestros pacientes, al menos uno de estos marcadores de laboratorio de mal pronóstico estuvo alterado.

El tratamiento de la amiloidosis cardíaca comprende el manejo de la insuficiencia cardíaca y la terapia específica para la enfermedad amiloide subyacente. En el manejo de la insuficiencia cardíaca, el mantenimiento de un estado de euvolemia es fundamental, por lo que la restricción de sodio y el uso juicioso de los diuréticos son importantes. ^(^^9^^,^^12^^)^ En nuestro estudio, el 100% de los pacientes recibieron diuréticos. Debido a que el gasto cardíaco depende de la frecuencia cardíaca, y debido a la tendencia a la hipotensión ortostática, los antagonistas neurohormonales como los betabloqueadores e inhibidores de la enzima convertidora de angiotensina (IECAs) no son efectivos ni bien tolerados. ^(^^9^^)^ En nuestro estudio el 50% (4 casos) de los pacientes recibió betabloqueadores, IECA o antagonistas de los receptores de angiotensina II (ARA-II) al inicio. En pacientes con fibrilación auricular, es posible que sea necesario utilizar betabloqueantes para el control de la frecuencia. Los bloqueadores de canales de calcio no dihidropiridínicos se unen ávidamente a fibrillas amiloides y están contraindicados debido al riesgo de hipotensión profunda y síncope. La digoxina generalmente se evita por mayor riesgo de toxicidad, y en la amiloidosis ATTR está contraindicada. ^(^^9^^,^^16^

El tratamiento específico en amiloidosis ATTR se basa en tres puntos importantes: supresión de la síntesis de TTR (trasplante hepático o silenciadores genéticos), estabilización de TTR (tafamidis, diflunisal o tolcapona) y eliminación de los depósitos (doxiciclina, doxiclina-ácido tauroursodesoxicólico (TUDCA) y otros). ^(^^16^^)^ El trasplante hepático, ha sido empleado en ATTRm, como una manera de eliminar la principal fuente precursora de TTR. La supervivencia reportada en pacientes con mutación Val30Met y clínica predominantemente neurológica es superior al 50% a los 20 años. ^(^^16^^)^ El caso 1 presentó esta mutación genética (que es coincidentemente la más frecuente reportada fuera de Estado Unidos por Maurer *et al.*), ^(^^28^^)^ se le realizó un trasplante de hígado, habiendo recibido tafamidis y doxiciclina desde el diagnóstico y actualmente es el paciente con la mayor sobrevida.

En amiloidosis AL el tratamiento de primera línea más utilizado consiste en una combinación de 3 fármacos: ciclofosfamida (agente alquilante), bortezomib (inhibidor del proteasoma) y dexametasona, que se administra semanalmente. ^(^^9^^)^ Se ha reportado que estas terapias modernas causan remisión y prolongan la supervivencia. ^(^^9^^,^^12^^)^ En el caso 6, solo toleró un ciclo de esta terapia triple asociada a rituximab, como terapia específica por la macroglobulinemia de Waldenström. A el caso 5, debido a que presentaba mieloma múltiple, se le indicó talidomida en lugar de ciclofosfamida y pamidronato, sin respuesta favorable. Es importante mencionar que luego de iniciada la quimioterapia para amiloidosis AL, el pronóstico depende de la respuesta hematológica. ^(^^9^

El trasplante cardíaco se ha planteado en amiloidosis AL para pacientes con insuficiencia cardíaca avanzada y una buena respuesta hematológica a la quimioterapia inicial, seguido de un trasplante autólogo de células madre; sin embargo, requiere de centros especializados para lograr una supervivencia aceptable y baja tasa de complicaciones. En amiloidosis ATTR, el trasplante tiene un rol menor, debido a la afección multiorgánica de la ATTRm y la edad avanzada en la ATTRwt. Se ha planteado en casos seleccionados el trasplante combinado de corazón e hígado de manera secuencial. ^(^^9^^,^^16^

Finalmente, las causas más comunes de muerte son la progresión de la insuficiencia cardíaca y la muerte cardíaca súbita. ^(^^9^^)^ La causa más común de muerte súbita es secundaria a la disociación electromecánica que resulta en actividad eléctrica sin pulso, más que al desarrollo de arritmias ventriculares. ^(^^2^^,^^12^^)^ Esto corrobora lo encontrado en nuestro estudio, donde la causa de defunción al año en todos los casos fue muerte súbita.

## Conclusión

La amiloidosis cardíaca es una enfermedad poco diagnosticada en nuestro país, pero no por ello poco frecuente. En esta serie de ocho pacientes las manifestaciones clínicas más frecuentes fueron la insuficiencia cardíaca y la neuropatía sensitivo-motora. La mortalidad fue del 25% al año, y en todos los casos como muerte súbita.

## Financiación:

Ninguna
